# The Hospital. Nursing Mirror

**Published:** 1898-06-25

**Authors:** 


### "The Hospital" Nursing Mirror.

Being the Nursing Section of "The Hospital."

[Contributions for this Section of "The Hospital" should be addressed to the Editor, The Hospital, 28 & 29, Southampton Street, Strand, London, W.C., and should have the word "Nursing" plainly written in left-hand top corner of the envelope.]

### News from the Nursing World.

### FOR THE LONDON HOSPITAL.

Great preparations are in progress for making a great success of the Press Bazaar which is to be held at the Hotel Cecil on Tuesday and Wednesday, June 28th and 29th, on behalf of the London Hospital. The bazaar will be opened by H.R.H. the Princess of Wales, and the number of personages well known in society who will take part in the proceedings will be very large. The stall to be held by the London Hospital itself will no doubt attract many friends, and, of course, all the nurses are very enthusiastic about it. Our own stall should certainly prove a feature, as the Prince of Wales himself is a contributor to it, having sent a supply of fruit to The Hospital Stall, to be sold in aid of the London Hospital. The stall will be charmingly decorated through the kindness of Mr. Piper, the well-known florist of Bishop's Road, Paddington. We hope our readers will not forget to send contributions to us, however small.

### DEATH OF A CRIMEAN NURSE.

Last week there died at Berwick a nurse who had served under Florence Nightingale in the Crimea. Her name was Mrs. Margaret Whittaker, and her husband was a sergeant in the 33rd Regiment, who fell in action. Mrs. Whittaker attended the wounded after the battle of Alma, and at the time of her death was in receipt of a Government pension for her services in that campaign.

### THE "DAY OF REST" AT HERTINGFORDBURY.

A large number of metropolitan nurses spent a "day of rest" at Hertingfordbury. Frequent services were held in the parish church by the Bishop of Thetford, assisted by the rector, Canon Burnside. Dinner and tea were provided for the guests, who filled up the unoccupied intervals in country walks.

### ST. MARY'S HOSPITAL, PLAISTOW.

At the Small Queen's Hall, Langham Place, W., a concert was given on the afternoon of Wednesday, 22nd, by Miss Clara Maisey (Medallist, R.A.M.), assisted by Miss Gertrude Hughes, Miss Margaret Godfrey, Miss Olga Nethersole, Mr. William Nicholl, Mr. Frederick Fredericksen, Miss Freida Calaminus, and Miss Bertha Callen. The proceeds were devoted to the needs of St. Mary's Hospital for Sick Children, Plaistow.

### INVALID CHILDREN'S AID ASSOCIATION.

A very successful drawing-room meeting to promote the welfare of the Invalid Children's Aid Association was held at 34, Grosvenor Square on Friday, June 10th, by the kind permission of Mr. and Mrs. Taylor, when the satisfactory sum of nearly £200 was collected, many wealthy and well-known members of society being present. The special feature of the work of this association is the befriending of invalid and crippled children of the London poor in their own homes by volunteer visitors.

### THE MATERNITY CHARITY AND DISTRICT NURSES' HOME, PLAISTOW.

Lady Battersea presided at a meeting held, by the kindness of Lady Rothschild, at 19, Grosvenor Place, to forward the work of the Maternity Charity and Nurses' Home at Plaistow in West Ham. Lady Battersea, the Rev. H. D. Burton, Miss Alice Ravenhill, Mr. H. Phillips, and the Bishop of Colchester addressed the meeting.

### COOKERY DEMONSTRATION.

The Committee of the National Refuges for Homeless and Destitute Children arranged a Cookery Exhibition at Sudbury Hall Girls' Home on the 22nd inst. After a visit to the kitchen and seeing the cookery class prepare a varied menu, the visitors adjourned to the dining hall and partook of the dishes. Mr. Edward Carlile (Master of the Cooks' Company) then distributed the prizes and awards. Music was provided by the girls themselves and the band of the Boys' Home at Twickenham. The home was thrown open to inspection, and tea and coffee served in the schoolroom.

### A NEW POST.

Miss Beatrice Cutler, who was trained at St. Bartholomew's Hospital, and for the last seven or eight years has held the post of sub-directress of the Girls' Medical School in Cairo, has been appointed inspector of the female pilgrims at Tor, by the Egyptian Quarantine Board. On their return from Mecca this year the pilgrims are put into quarantine in camp for twelve days, where they are thoroughly inspected before being allowed to return to their several countries. Miss Cutler is the first English lady who has held this post, which this year has become such an important one on account of the plague in India. Her knowledge of Arabic and of the native characteristics will doubtless be of great service to her during her two months of exile in camp life at El Tor.

### A CHILDREN'S GUILD IN DUBLIN.

A children's guild has just been started in Dublin, under the title of the "Moy-Mell Guild," with the excellent object of encouraging the children of the rich to take a personal and helpful interest in the sick children of the poor. The inaugurating meeting was held the other week at the Children's Hospital, Temple Street, over which the Countess of Fingall presided, and rules were drawn up and adopted. Members pay an entrance fee of 6d., girls supplying two garments and boys two toys each year. They also make collections, by which it is hoped to endow a Moy-Mell Guild cot at the Children's Hospital, Temple Street. Mrs. Tollerton, secretary of the Philanthropic Society, explained the system upon which it was proposed to work the Guild, beginning in Dublin, and afterwards extending the movement throughout Ireland, and even further afield. A number of ladies then formed themselves into a provisional committee, amongst whom were the Countess of Fingall, Lady Longford, Lady Martin, Mrs. James

Talbot Power, Lady Maurice, Mrs. P. J. Plunkett, and several others; and a sub-committee was also appointed, upon which Miss McGivney, the lady superintendent of nurses at the Children's Hospital, has a place, to draft the working details of the scheme. The "Moy-Mell Guild," if it succeeds, as it should do, will, like Lord Winchelsea's "Order of Chivalry" for children in England, be doing good educational service.

### LOUGHBOROUGH NURSES.

By the kindness of an anonymous donor the Queen's Nursing Association was formed last year, and a nurse was engaged to attend the poor of Loughborough. The nurse is most popular and hard working, but cannot meet all the demands on her time. Consequently, the association have endeavoured to obtain a grant from the board of guardians to enable the services of a second nurse to be secured. As the country members, however, hold the preponderance of votes on the board, and this is a matter that only benefits the borough, the grant was not carried. The association has increased the nurse's stipend to £85 a year, and she attends as many parish cases as possible. Amongst the items of expense we note that of the purchase and repair of a bicycle for the nurse's use. Evidently, therefore, the Queen's Nursing Association has recognised the economy of the bicycle, and accepted the responsibility of keeping it in order.

### CEYLON NURSING ASSOCIATION.

A general meeting of the Ceylon Nursing Association was held at Adam's Peak Hotel on May 7th. The work of the society is increasing, its position satisfactory, and endeavours are being made to extend its membership. Mrs. Pole Carew, who has been hon. secretary during the past year, resigned, as she was leaving the island, and Mrs. L. P. Fisher was elected in her stead. Resolutions adding R. 15 per week to the charges made to non-subscribers; declaring that R. 100 should be the subscription of a life member, and of R. 150 that for family membership, that moneys thus received should be regarded as capital, and that monthly nurses should not be booked for more than six weeks at a time, were passed. Mrs. Pole Carew was elected life-member of the association and honorary member of the executive council.

### NURSES' FEES TO MEDICAL MEN.

The generosity of medical men towards the sick poor, be they nurses or not, prompts an immediate return of reciprocal services to the families of the poorer professional men when illness falls to their share. The case of Dr. Jones, in the *Lancet* for the 18th inst., is one in point. His wife falling ill of typhoid, he asked a nursing institution if he could have nurses at reduced terms, and found that he would have to pay £3 3s. each. Another institution, however, was more complacent, and expressed pleasure in taking lower fees from medical men when it could be afforded. The high fees of trained nurses render them a luxury even for the rich—amongst whom the majority of doctors cannot be counted—and it seems hard at first sight that a profession that gives so freely should not as freely receive. But the cases are not analogous; the medical man can attend a score of patients in a day, the nurse must devote her time and strength to one. If one patient cannot pay his doctor another can, but the nurse cannot "cut" her loss in this way. The immense majority of the middle-class cannot afford a trained nurse in sickness, and most of the trained nurses cannot afford to give their help even to their best friends, the doctors. This is a dilemma out of which there is no apparent exit. Many leaders of the nursing world aim at making the training of a nurse more difficult and expensive, maintaining that thereby they improve the status and earning power of nurses. But in the meantime, the greater number of persons who need their services have lightened purse and heavier burdens. Can it be that the heyday of the amateur nurse is approaching?

### THE WIMBLEDON CARNIVAL.

The Surrey Convalescent Homes for Men and Women have benefited to the extent of £200 by a carnival which took place at Wimbledon early in the month. The idea of organising a fête in connection with the opening of a new street originated with a working man, and was eagerly taken up by the community. A handsome prize fund was subscribed, attractive displays arranged, and plenty of music provided. Colonel Mitchell's grounds at Cannizaro were thrown open and thronged. Had fine weather continued, the success would have been still more marked.

### ACT FIRST; ASK AFTERWARDS.

Of course, everyone knows that the easiest way of getting a thing done is to act quietly first and to talk about it afterwards. It is not a little amusing, however, to find this rule which answers so well in private life, translated into official practise. The Hampstead Board of Guardians appealed to the Board of Trade for permission to lay out tennis courts for the nurses and officials of the workhouse, and upon receiving a guarded approval, one of them said, "Now we can get to work," to which the clerk replied, "Its done, Sir!"

### A PENAL OFFENCE.

We have received a most amusing postcard, which, as our correspondent sends neither name nor address, can hardly be treated as serious, therefore we invite our readers to share the joke. "As the appellation of 'nurse' is so much abused by illicit 'massage' proprietors," she writes, "may I suggest that it should be rendered a penal offence for anyone to describe herself as 'nurse' unless possessing a qualifying certificate, such certificate to be cancelled if the person holding the same is found to be working at or conducting one of these establishments, or for any other gross misconduct. I ask that this be done for the protection of *bonâ fide* nurses." Unfortunately, although our correspondent has given the word, it cannot be done. If it were possible to render the act of calling oneself a nurse without a formal certificate a "penal offence," who is going to hunt up the thousands of innocent offenders on this score, and consign them to prison cells? Which and whose certificate also, we should like to know, would be regarded as qualifying, and who would have the right to cancel them, if abused? And, lastly, how are *bonâ fide* nurses to reap any advantage if all this were done? No one who wants a nurse goes to "illicit" massage establishments for her, and if these establishments are illicit, why does not the law intervene and consign the proprietors to penal servitude? When our correspondent has answered all these points to the general satisfaction of the majority of nurses, she will have solved some of the knottiest points in nursing politics.

### SHORT ITEMS.

By an error in last week's issue the name of the assistant superintendent of the Irish Branch of Queen Victoria's Jubilee Institute for Nurses was given as Miss Blackman instead of Mrs. Blackmore.

### Post-Graduate Clinics for Nurses.

BY A TRAINED NURSE.

### ARTIFICIAL METHODS OF FEEDING.

IV.—SUPPOSITORIES—HYPODERMIC AND NASAL FEEDING.

PATIENTS fed entirely by enemata usually complain—especially for the first forty-eight hours—of extreme thirst. In suitable cases the medical man may allow very small quantities of water by the mouth, or in cases where this is not permissible he may instruct the nurse to give once in the twenty-four hours an injection of a pint of warm water, to which a few grains of salt should be added. Constant swabbing of the mouth helps to relieve the thirst so often accompanying rectal feeding.

It will be necessary in order to complete the list of methods of feeding by rectum just to touch on nutrient suppositories, though nutrient enemata have to a very large extent supplanted them. These are used in those cases where it is only necessary to feed the patient by artificial means for twenty-four or forty-eight hours, and when some other condition renders it important that the patient should not be disturbed so much as is entailed in the giving of enemata. In the hæmatemesis of gastric ulcer suppositories are employed, since the patient does not need much feeding, and quiet is essential. Sometimes, too, in the unconsciousness accompanying hemiplegia suppositories may be used for a day or two just because they are so easily applied.

In some gynæcological cases it is found that the tight tamponing of the vagina interferes with the absorption of rectal injections, and suppositories may be preferred in such cases as being smaller in bulk. Home-made suppositories are not only most troublesome in the preparation, but the amateur is not able to make them so scientifically nutritious nor so consistent in substance. After well oiling these—and having seen some nurses use glycerine for this purpose, reminds me to point out that glycerine will most surely prove irritating and lead to their expulsion—the nurse, while the patient lies on the left side, should push up the suppository into the rectum slowly and gently by means of a soft bougie—if left low down so soon as the suppository melts some of its substance is liable to escape.

Feeding by hypodermic is rare and unsatisfactory, and it is usually the doctor who administers nourishment in this form as a last resource in cases of collapse. Sometimes a hypodermic of alcohol has rallied and saved a patient, but nourishment given by this method has never proved of much value. In any case this and feeding by intravenous injection will not come within the province of the nurse, although it is quite within her province to be aware that such methods exist.

The injection of milk into the veins has been tried in the case of cholera patients and some others, though this has been superseded by salt water injections, which are said to fulfil the same purposes of stimulation and rallying.

Nasal and stomach-tube feeding come within the domain of the modern nurse, and some practical points on these—as well as on some more simple methods whereby patients can be induced to take food—will be given.

Some patients retain the power of swallowing though they may have lost all power of suction. Food may then be given by means of a bent glass tube, the nourishment being either gently syringed or poured from a feeder into the tube. It is important to carry this out cautiously, to avoid introducing the food while the patient is taking in his breath, and to allow plenty of time between "swallows." If the patient keeps the teeth clenched food can be given by means of a medicine-dropper, though by this method, unless the amount is supplemented by rectal injection, the patient will not take nearly enough food.

In every form of feeding—artificial or otherwise—patience will overcome the gravest obstacles, and a nurse who is properly impressed with the value of "kitchen medicine" will never tire even through the most tedious processes of feeding.

The nurse must try every means of inducing a patient to take sufficient nourishment, and it is surprising how tact and perseverance will overcome the most obstinate patients and the most fractious children. Many sick children refuse food, especially if the mouth or throat is sore and swallowing proves painful. In diphtheritic and scarlet fever throats all attempts to make quite young children swallow are resisted, and nasal feeding must be resorted to. Older children with bad throats may often be induced to take food if the nurse will give them somewhat large pieces of ice and instruct them to hold these rather far back in the throat. After two or three pieces of ice have been thus melted in the throat, a temporary anæsthesia by freezing may be established which will last long enough to enable the patient to swallow painlessly a fair amount of liquid food. Sometimes, too, the doctor will order a cocaine solution to be painted on the throat before nourishment is given by the mouth. Several doctors for whom I have nursed have asked me to try the feeding of unconscious children by means of fluid nourishment poured into the nostril from a spoon. In some cases, where the teeth are clenched or there is difficulty in swallowing, food may be given thus, the child being placed with the head rather far back on the pillow and the spoonsful given very carefully.

In cases of unconsciousness less danger of choking results from this method than from attempting to feed by mouth, and any excess of fluid tends to run out at the other nostril. But I must confess to an infinite preference for nasal feeding by tube to this tedious and somewhat unsatisfactory process. After a little experience one becomes very adept at nasal "spoon feeding," but it takes a very long time to thus administer enough to count as "a meal."

I think most nurses will agree with me in a fondness for the nasal tube feeding of children. It can be done with so little fuss or trouble, and it is such a satisfaction to see a diphtheritic child receive the generous supplies of milk, cream, koumiss, and whipped egg, which can be given so easily by this method.

A baby with sore mouth is thus most beautifully nourished, as also are persons whose mouths are excoriated from the taking of corrosive poisons. The insane and the choreic, or the obstinate child who persistently bites the stomach tube, may be fed nasally *nolens volens*. After pharyngeal operations causing trouble in swallowing, in tetanus, or spasm of the throat muscles, the nurse feels so very confident that she can keep up her patient's nutrition by the two satisfactory methods at hand—nasal or stomach-tube feeding.

### Presentation.

MISS ELIZABETH WALDEN, who has held the post of Queen's Nurse at Wribbenhall, Worcestershire, for the past five years, was presented by her committee on leaving with a handsome travelling clock and a case of scissors as parting gifts. Miss Walden is now working as Queen's Nurse with the Victoria Nurses' Association, founded last year at Huddersfield.

### Where to Go.

QUEEN CHARLOTTE'S LYING-IN HOSPITAL, MARYLEBONE ROAD, N.W.—The foundation-stone of the Nurses' Home of Queen Charlotte's Lying-in Hospital, in the Marylebone Road, will be laid by Viscountess Portman on the afternoon of Wednesday, July 6th.

### Training in the Provinces.

In a series of articles published in THE HOSPITAL some time ago full particulars were given of the training offered to nurses in the larger metropolitan training schools, with an account of the conditions under which the nursing work of the London hospitals is carried on. In the following series similar details will be given of the nursing arrangements in the principal training schools in the provinces and in Scotland, which will enable those wishing to take advantage of the excellent training to be had at many country hospitals to see for themselves at a glance on what terms and under what circumstances such training is obtainable. Sincere thanks are due to the matrons of the various institutions for the help they have rendered in supplying full information concerning rules and regulations.

### ABERDEEN ROYAL INFIRMARY (170 Beds).

### TERMS OF TRAINING.

Probationers are received at the Aberdeen Royal Infirmary for a term of three years' training, a six months' trial being required of candidates before they are finally accepted. The engagement includes a fourth year in the service of the hospital, when nurses who have satisfactorily passed their final examination become charge nurses or may be sent to private cases. A certificate is given on satisfactorily completing the engagement.

Candidates are expected to be fairly well educated. No salary is given during the six trial months. Probationers begin with a salary of £10 for their first year, rising the second year to £18 and the third to £20, after which, as charge nurses, they receive £25 per annum. Laundry expenses and indoor and outdoor uniform are provided by the hospital. Sisters are paid from £30 to £40 per annum, and are given indoor uniform.

Probationers are put upon night duty for periods of three months at a time. Lectures are given to the nurses by members of the medical staff.

### HOURS ON AND OFF DUTY.

Probationers are on duty in the wards from 7.30 a.m. to 2 p.m., from 2.20 to 5 p.m., from 5.15 to 7 p.m.; or from 7.30 a.m. to 2.20 p.m., and from 5.30 to 9 p.m. Time is allowed for lunch during the morning, twenty minutes for dinner, fifteen for tea, and from two to three hours off duty each day. In the medical wards, where the staff visit from 9 to 10 a.m., the nurses may have their two off-duty hours in the morning.

Charge nurses do not go on duty till 8.15 a.m.; otherwise their time-table is similar to that of the probationers. Sisters are on duty from 8.15 a.m. to 3 30 p.m., when, if work permits, they go off duty till 6.30 p.m. "Long Passes" are given from dinner on one day to 7.30 a.m. the next day. The night staff are on duty from 9 p.m. to 9 a.m., being relieved during the night for refreshment, which is served in the ward kitchen. There are two sisters on permanent night duty, and these have a night off once a month when possible and the wards are quiet. The nurses are on night duty for period of three months at a time.

### MEALS.

Meals for the day staff are all served in the dining-room. Breakfast is at 7 a.m., luncheon from 9.30 to 10.30 a.m.; dinner from 2 to 2 45 p.m.; tea, 5 to 5.30; and supper at 9 p.m. The night nurses have supper at 6.30 p.m., tea in the ward kitchen during the night, and dinner at 9.15 a.m.

The sisters dine in their own dining-room at 6 p.m. Weekly allowances of tea, sugar, butter, &c., are made to sisters and to nurses on night duty.

### BATH ROYAL UNITED HOSPITAL (130 Beds).

### TERMS OF TRAINING.

Candidates for training at the Royal United Hospital, Bath, must be between twenty-three and thirty years of age, a trial of two to three months being required before final acceptance. The certificate is given after passing an examination and satisfactorily completing a three years' engagement. A few paying probationers are taken, paying a premium of £30 for one year with the understanding that if approved they may remain if they wish for the second and third years' training on the same terms as the ordinary probationers. No difference is made between special and ordinary probationers. These latter are not paid during their first year. The salary given the second year is £12, and for the third £20. Staff nurses receive £25 a year and sisters £30, rising by yearly increments of £2 to £40. Laundry expenses are provided; indoor uniform is given the second year, and indoor and outdoor uniform the third and subsequent years.

Night duty is taken for two months at a time; day duty for four months or longer.

Lectures are given to the nurses by the Lady Superintendent (Mrs. Mathias) and the resident medical staff, and gold and silver medals are presented each year to the two nurses who do best at their final examinations and gain highest marks for general good conduct and work throughout the three years.

### HOURS ON AND OFF DUTY.

Day nurses go on duty in the wards at 7 a.m., leaving them at 9 p.m., with time allowed for meals and three hours off on alternate days, or daily when possible. Once a fortnight a pass from 2 to 9 p.m. is given. These hours are the same for all the staff.

Two weeks' holiday are allowed at the end of the first year, and three weeks the second and third year.

### MEALS.

All meals are served in the dining-room, except the night nurses' midnight meal, and the only "allowances" given into the nurses' keeping are the stores for night consumption.

Breakfast for the day staff is served at 6.40 a.m., lunch at 10, dinner from 1 to 1.45 p.m., tea at 4.30, and supper at 9 p.m.

### BIRMINGHAM AND MIDLAND EYE HOSPITAL (100 Beds).

### TERMS OF TRAINING.

Training in ophthalmic nursing is given at this institution, candidates for the post of assistant nurse or probationer being received after a satisfactory month's trial. Those probationers who have already trained in general nursing at a recognised school may be promoted to the post of charge nurse; those who have not had previous general training are promoted to become out-patient nurse or night nurse. There is in this hospital a ward of thirty beds for children, which provides an opportunity for nurses to study a variety of children's ailments, many infantile eye diseases arising from mal-nutrition and general ill-health. Lectures on ophthalmic nursing are given to the assistant nurses once a week by the resident medical officer.

Candidates for the post of charge nurse must be between 24 and 30 years of age, and have received three years' training in a general hospital. The salary given is £25 per annum. Laundry expenses are allowed, and indoor uniform is given to, and made for, the nurses. Four weeks' holiday is given in the year. Night duty is divided between the two out-patient nurses, each taking six months at a time, with the help of one probationer.

Hours On and Off Duty.

The probationers' working day is from 7.15 a.m. to 8 p.m., staff nurses from 8 a.m. to 8 p.m.; night nurses are on duty from 9.15 p.m. to 8.45 a.m. Every nurse has two hours off duty every alternate day, from 3 to 10 p.m. on one day a week, and alternate Sundays from 10 a.m. to 2 p.m., or 2 to 10 p.m., with extra time when the work is light. From 2 to 6 in the afternoons is usually a very light time in the wards.

Meals.

Meals are served in the duty room off the day room, the hours being as follows: Breakfast, 7.30; lunch, usually 10.45; dinner, 1.30 p.m.; tea about 4 p.m.; and supper at 8 45. Butter is given out in daily allowances to the nurses, other stores once a week.

### Florence Nightingale on Nursing.

The following letter from Florence Nightingale to Lady Aberdeen on the subject of nursing is full of interest and encouragement. It is dated May 5th, 1898.

"Dear Lady Aberdeen,—I do rejoice at the success which has attended your efforts to initiate the plan for establishing trained district nurses in Canada. With great interest I have read the papers you have so kindly sent me. Let me gladly add myself as a witness of experience here to the great blessings which the trained district nurses have been to the sick poor.

"If you are able to maintain the high standard for your nurses which you have laid down, and succeed in attracting good young women to enter upon the work, there can be no doubt that it will go on and prosper. Difficulties and trials there must be, but with so noble an object it is worth the expenditure of much labour and patience.

"What has been the experience of the last thirty years with regard to the improvement of hospital training and the means by which it has been attained? This, namely, that it has been brought about, first, by making the hospital a 'home' fit for good young women—educated young women—to live in and pursue their calling, and, next, by raising the character of nursing into a genuine calling by which nurses can earn an honourable livelihood. Then from the hospital training school the area of the trained nurses' work became extended to private nursing—nursing the well-to-do—and latterly to that far more numerous class of patients who are either entirely destitute or only able to make a small contribution for the services of the nurse, and yet who are not fit subjects for hospital treatment.

"It is especially, and above all, to this last class that the trained district nurse has proved so great a boon. For the duties of a district nurse more experience, more self-denial is wanted than for those of a hospital nurse or private nurse, who have the doctor always at hand to refer to, and have all the appliances of hospital or home at the service of the patient.

"The success of district nursing depends more than in hospital and private nursing upon the character of the nurse; and the character of the nurse depends very much upon the nature of her training, and the continuance of those helps, physical and moral, which the good hospital 'home' has supplied to her.

"These helps have been found in the system of district nurses' homes under trained superintendents which have been established here with so much success in London, Edinburgh, Dublin, and other large towns, and which you propose to adopt in Canada. Is it not to these homes that you will have to look to train in district work and qualify for service in small towns and country places—pursuing their calling under periodical supervision, and as members of a society inspired by the *esprit de corps* of joint workers in a noble and Christian cause? No doubt in some respects your population, especially in rural districts, differs much from that of an old country, and somewhat different methods will be required. Happily there does not exist with you that large number of sick poor who are unable to pay anything for the services of the nurse.

"Heartily do we wish success to the Victorian Nurses, and to all Canadian workers in this good cause."

### Minor Appointments.

Mogden Isolation Hospital, Isleworth, Middlesex.—The following appointments were made on the 20th inst.: Charge Nurses—Miss Alice Fletcher, who received three years' training at Halifax Infirmary, and has since held appointments at the Sheffield City Hospital; Miss Elizabeth Jackson, who received three years' training at Birmingham Infirmary, and has since held the appointment of charge nurse at the Eastern Fever Hospital, Homerton; Miss Lilla Snow, who received three years' training at St. George's Infirmary, Hanover Square, and has since had charge of wards at Croydon Borough Hospital. Assistant Nurses—Miss Harriette White, who was trained at the Kent and Canterbury Hospital, and has since held appointments at the Bromley Fever Hospital and at the City Hospital, Birmingham; Miss Ellen G. McKendrick, who was trained at the Delancey Fever Hospital, Cheltenham, and has since held appointments at Borough of Bootle Corporation Hospital, Linacre Lane; at City Hospital South, Grafton Street, Liverpool; and at Stroud Road Hospital, Gloucester.

Brentford Union Infirmary, Isleworth.—On June 15th Miss H. Elliott, who has been sister at the Rotherham General Hospital and at the Birmingham General Hospital, was appointed Assistant Matron of this infirmary. She was trained at Addenbrooke Hospital, Cambridge. On the same day Miss Millicent Parker was promoted to the post of Sister in succession to Miss E. Todd, who has just been appointed Sister in the Army Nursing Service. Miss Parker holds a three years' certificate from the Leamington and Warnford Hospital, and for the last two years has been ward nurse of the above infirmary.

South Stoneham Union Workhouse, West End, near Southampton.—Miss Meacham has been appointed Working Superintendent Nurse of this institution. She was trained at Sunderland Workhouse Infirmary, and holds a three years' certificate for general training, fever, and midwifery (L.O.S.) She has since been charge nurse at the Towns Hospital, Glasgow, and engaged in private work.

Great Western Railway Hospital for Accidents, Swindon.—Miss E. M. Boutell has been appointed Surgical Nurse of this hospital. She was trained, and afterwards principal night nurse for five years, at the Infirmary, St. John's Hill, London, S.W.

Wantage Cottage Hospital.—Miss Alice L. Dallimore has been appointed Matron of this hospital. She was trained at the West Ham Hospital, Stratford, E., and was afterwards staff nurse and sister of women's and children's wards there.

The Royal Infirmary, Windsor.—On May 26th Miss C. S. White, who was trained at the Durham County Hospital, was appointed Charge Nurse of this infirmary. Miss Whyte has been staff nurse at Kendal Memorial Hospital.

Fever Hospital, Thorp, Easington.—Miss Annie Cooper has been appointed Nurse-Matron of this hospital. She has lately held the post of charge nurse of the women's ward of the Hartlepool General Hospital.

### Novelties for Nurses.

### A GOOD FURNITURE POLISH.

Ronuk is a polishing paste, used with equal success both on wooden and leather articles. On woodwork this paste leaves a fine hard polish, from which dust and other matters can be thoroughly and easily removed. We consider Ronuk is especially adapted for hospitals and other buildings where all woodwork, &c., is kept in a strictly sanitary condition. London depôt, 83, Upper Thames Street, E.C.

### The Matrons' Council.

THE final session of the Matrons' Council was held on Thursday afternoon, June 16th. Mrs. BEDFORD FENWICK read a paper on "A Practical Standard of Nursing," in which she sketched out plans for teaching, certificating, and registering nurses. She desires to see the portal to the nursing profession made so small that only the best women have a chance of entering. For this reason she approves of the preliminary training introduced by Miss Strong at Glasgow and improved upon by the London Hospital Preliminary School at Tredegar House. Her curriculum orders the three years training as follows: For 18 months the students are to be probationers and to have their time apportioned out for them as in a boarding school. The second 18 months, when they are recognised as staff probationers, to be spent in special wards, a three months' spell being devoted to each. Frequent school examinations test the progress of the student, who is finally to be examined by a council composed of medical men and matrons appointed by the State. The student then becomes staff nurse and advances according to her opportunities.

Miss ISLA STEWART, in commenting upon the paper, remarked that she had always desired State registration for nurses, and for that reason had accepted the chairmanship of the Council. Whilst, however, Mrs. Bedford Fenwick was an enthusiast she was a practical person, and although on paper the sketch of training nurses was excellent, she considered the difficulties insurmountable. In the first place, the matron was the servant of the hospital, and her first duty was to nurse the sick. Training the nurses came second, and it seemed impossible to put the teaching of nurses first without sacrificing the patient's interest. With double the staff, and nurses machines it might be done, but nurses were human, and the matron was constantly adjusting and readjusting the work. The next difficulty was that of finding sisters who could and would teach. She did not consider teaching their duty. There was an enormous mass of knowledge to be assimilated, and the probationers ought to pick it up and assimilate it. There were a number of qualities that could not be taught in a hospital ward, such as tact and personal influence, as well as other qualifications, such as a knowledge of housekeeping, storekeeping, book-keeping, cookery, and so on.

Miss POOLE asked if State registration did not involve a public examination?

Miss MOLLETT explained that a State certificate could only guarantee that the nurse possessed the technical knowledge necessary for her profession. She asked if it were fair to the medical staff to ask them to give so many lectures without remuneration? Miss PELL-SMITH also asked if it were possible for the nurse to devote so much time to theoretical and practical work without neglecting their patients?

Miss HUXLEY said that in Dublin five hospitals combined and paid a lecturer to instruct the probationers. Later on in the discussion, she said that the society was called the Dublin Metropolitan Technical School for Nurses, that candidates had to pass an entrance examination for it before being admitted as probationers into any of the five hospitals; that each student paid £1 towards the expense; and that the weekly lecture delivered on Monday was repeated again on Thursday, thus permitting all to attend. At the termination of the course certificates were granted by both hospital and school, the one being of no value without the other. Between 50 and 100 nurses attend.

Mrs. BEDFORD FENWICK said that medical men ought to be paid for giving lectures to nurses. They were well paid at St. Bartholomew's for doing so. In her opinion a sister's duty in the wards was to make her subordinates do the work, teaching them the while. That the nursing school should be as much a part of the hospital as the medical school. She quoted the instances of Middlesex and London Hospitals voting sums of public money to place their medical schools in a financially sound position. If this were so, why should not the nurses have a slice? Miss Gordon, of St. Thomas's, she believed, received a good salary as superintendent of the Nightingale School, in addition to her stipend as matron of the hospital.

Miss ALICE WADMORE then read her paper, "Need of Sanitary Knowledge for Nurses," which was followed by Miss SCOTT's paper on "How to Meet the Increasing Difficulty Experienced by Matrons of Hospitals of under forty Beds in Procuring Probationers." The paper and the discussion merely opened a subject of enormous difficulty, the feeling being that probationers could not be satisfactorily trained at small hospitals, and that the objection shown by the larger hospitals to partially trained probationers made the time passed as such useless. In the meanwhile as small hospitals must be nursed, and were too poor to pay an adequate staff, the matron had often to be the whole staff in one.

The session terminated after passing a resolution inviting the hon. members of the society to attend the quinquennial meeting of the International Council of Women to be held in London next summer, and offering them hospitality for the conference week.

### The Bangor Hospital Inquiry.

AT the last monthly meeting of the Bangor City Council a report was presented by a sub-committee which had been appointed to investigate certain charges arising out of the death of a boy in the Borough Hospital. The sub-committee are of opinion that the fact that the matron allowed the child in question to be taken into the open air without the direct permission of the medical man in attendance "did in no way, or in the remotest degree, conduce to the subsequent relapse and eventual death" of the patient. With regard to the food the evidence was contradictory, "but it was not proved to our satisfaction that the food was deficient either in quality or in quantity. We would, however, recommend that a nurse be present at all times when meals are served to young patients."

In regard to night nursing, they are satisfied that "a nurse is always present either within the ward or in the adjoining duty room." As to the action of the matron and nurses, the sub-committee say: "We find that the charge of want of tact on the part of the matron has not been established; but Mr. and Mrs. Downs were naturally so much distressed in view of the relapse of their child that their feelings were easily wounded, and the matron may not at all times have exhibited a perfect evenness of temper. In regard to the charge that Nurse Jones slept at her post whilst on night duty, we find there is no evidence to support such a charge. In conclusion, we beg to recommend that the Hospital Committee be instructed to ascertain whether the regulations now obtaining at the hospital may be advantageously revised in the light of the experience gained since the hospital was opened. We wish it, however, to be understood that this recommendation conveys no reflection on the matron and the nursing staff."

After a long discussion the report was adopted.

### Death in Our Ranks.

MISS M. B. VICKERS, superintendent of the Bangor Institute for Trained Nurses, died on June 14th after a brief illness. She was the daughter of Mr. William Vickers, of Nottingham, and was trained at the Royal Infirmary, Chester, after which she was appointed head of the nursing department of the Deaconess Home in the same city. She then became superintendent of the Bangor Institute, where she carried out her duties with success. Much regret is expressed at her loss, and amongst the large number of wreaths sent was one from the president of the institute, Lady Penrhyn.

### Everybody's Opinion.

[Correspondence on all subjects is invited, but we cannot in any way be responsible for the opinions expressed by our correspondents. No communication can be entertained if the name and address of the correspondent is not given, as a guarantee of good faith but not necessarily for publication, or unless one side of the paper only is written on.]

### NURSES' AMBITIONS.

"AUSTRALIA" writes: In looking over "C. W.'s" letter of April 2nd, I am sure every reader must admire her ambitions. If "C. W." works in the same spirit as she writes, with a sympathetic heart and a light hand, she must make a good nurse, but no one should go into the nursing world puffed up with their own greatness. When I entered a London hospital I was ignorant in every way. I had never seen a hospital or a nurse. The little knowledge I had came from reading extracts of Miss F. Nightingale. I had one great craving ambition, to become a good hospital nurse. I made up my mind to give strength, talents, in fact, my whole heart. I did my best to observe, be obedient to my superiors in office; and with all my ignorance I have not failed as a nurse or a matron. I look back upon my early nursing days with pleasure.

### NURSING IN MILITARY AND NAVAL HOSPITALS.

WE have received a communication from a correspondent signing herself "Indignant," making grave charges against the manner in which the sick are treated in military and naval hospitals. She declares that they are insufficiently fed, deprived of necessary comforts, and without nurses. We have taken some trouble to inquire as to the correctness of her statements, and have heard from one who has been a patient in more than one naval hospital that although there is room for improvement in many ways "Jack" is made as comfortable as possible when sick. He remains on full pay, has trained nurses to superintend the nursing, and is provided with everything the doctor orders. When convalescent he receives his ordinary "rations," which are cooked for him, and which he can supplement with what he wishes, as he does when on duty.

### NURSES FOR PRISON SERVICE.

THE WARDEN OF THE ROYAL VICTORIA HOME, Bristol, writes: Earlier in the year I remember you referred in a leading article to nurses wishing to enter the Prisons Service. Will you permit me to call attention to the vacancies on the resident staff of this home as offering all the opportunities for doing good the former service presents, and to ask those who seek such work to refer to our advertisement in another column? I know of no position in life that offers more hourly possibilities for doing good than that of a sister here. Women of all classes, under sentence of imprisonment and otherwise, come under their care. Those who join us find all they can want in the direction referred to, and, of course, everything in the way of board, washing, and uniform found, a separate bed-room, and a sufficient stipend. May I hope you will see your way to give publicity to this letter, and so help both those who seek such work as ours and ourselves who seek their assistance?

### THE PLAGUE NURSE AND HER MONEY DIFFICULTIES.

"ON PLAGUE DUTY" writes: The monetary position of the nurse on plague duty seems to have depended so far entirely on the station to which she has been posted. In some stations she has been under English supervision; in others, under native officers, and, from five months' personal experience of the latter, I can vouch for the money difficulties of the latter position. As you say, we are entitled to free quarters, but I had to fight the question with my native "superior" as to whether the furnishing was to be paid by me or the municipality. Then, when the question was settled in my favour, I and the other lady to whom the nursing was entrusted, found ourselves continually obliged to expend considerable sums on "further" and "necessary" household articles. The relative position of a Poona and a Surat nurse can be seen at a glance in the following columns:—

| POONA. | SURAT. |
|---|---|
| 1. A number of nurses living under a superintendent, paying an inclusive monthly amount per head. Quarters in a European bungalow, comfortable, and suited to the needs of Englishwomen. | Two nurses "accommodated" in some rooms in the native High School. One of these "rooms" being a gallery round the central hall, required a fair expenditure in the matter of bamboo hangings to render it habitable for Englishwomen. |
| 2. Bedding and household linen provided. | Bedding and house linen provided by the nurse herself. |
| 3. All household and cookhouse utensils provided. | Additional crockery and supplementary cooking vessels had to be provided by nurse. |
| 4. Water free. | Monthly expenditure of Rupees 12 (16s.) on water. |
| 5. Cooking and attendance came under inclusive charge. | Necessity of providing cook, kitchen boy, table boy, sweeper, &c. |

These few particulars alone will suffice to show how very different the position of the nurse may be in a financial sense. I am not "complaining," because I have been very happy in my work and surroundings; but most certainly have I been worried over the money matters. One should always have a reserve by one in case of transfer, when plenty of ready-money is necessary for travelling expenses. These expenses are not recovered without a considerable lapse of time and a wearying correspondence. My experiences are of a native town without the wholesome supervision of a plague committee, composed for the most part of English gentlemen. I have had the pleasure of meeting the courteous city magistrate for Poona, and he has told me that he himself personally and repeatedly inquired into the comforts and requirements of the English nurses stationed at Poona, and very gratifying the results were. Very different were the ideas of my native "superior officer" as to an Englishwoman's ideas of sanitation and decency. Yet he had visited England; but I am afraid his inherent devotion to the accumulation of rupees blinded his mental and moral vision. Truly have I realised the words of an Englishman fourteen years resident in India: "The position of a white man in India without money is terrible; that of a white woman too awful to contemplate." Yet that has sometimes been my painful experience whilst under native officialism.

### The Society for the Prevention of Cruelty to Children.

ON Saturday, June 18th, a meeting was held in the old Henry VII. Hall at Fulham Palace, when the work and prospects of the Society for the Prevention of Cruelty to Children were discussed. Bishop CREIGHTON presided, and after his opening remarks, in which he warmly recommended the society to the notice of the public, not only on account of the children, but also on account of the great good done to the parents by means of the restraining influence exercised upon them, the Duchess of Somerset gave, in a quietly impressive way, some details of the work carried on throughout the United Kingdom, which showed how very necessary the society is, and how, in the most unsuspected places, cases of gross cruelty are detected and arrested. The Rev. B. Waugh was not able to be present on account of ill-health. The arduous work and continued strain of responsibility on account of the society have so told upon him that the doctors have ordered him complete rest for three months. The finances of the society are now in a fairly satisfactory condition, although still greatly dependent on the public for voluntary help. New inspectors have to be appointed, for at present the number of these very necessary officials is most inadequate to the population of the districts they supervise. The meeting was well attended, and the announcement that the work of the society was growing in the public estimation seemed to be borne out in the present instance by the well-filled hall and by the kindly interest taken in the proceedings by all who were present.

### For Reading to the Sick.

THE DESTINED UNITY.

**Verses.**

LIKE a mighty army moves the Church of God!
Brothers, we are treading where the saints have trod;
We are not divided, all one body we,
One in hope and doctrine, one in charity!
—*Baring-Gould.*

One God! one law! one element!
And one far-off, divine event
To which the whole creation moves!
—*Tennyson.*

Whatsoever spark
Of pure and true in any human heart
Flickered and lived—it burned itself towards Him
In an electric current, through all bonds
Of intervening race and creed and time,
And flamed up to a heat of living faith
And love, and love's communion, and the joy
And inspiration of self-sacrifice!
And drew together in a central coil
Magnetic, all the noblest of all hearts,
And made them one with Him, in a live flame—
That is the purifying and the warmth
Of all the earth. —*H. H. King.*

Elect from every nation,
Yet one o'er all the earth—
Her charter of salvation,
One Lord, one Faith, one Birth!
One Holy Name she blesses,
Partakes one Holy Food,
And to one hope she presses
With every grace endued. —*Stone.*

They whom many a land divides,
Many mountains, many tides,
Have they with each other part?
Have they fellowship in heart?

With each other join they here
In affliction, doubt, and fear;
That hereafter they may be
Joined, O Lord, in bliss with Thee.
—*J. M. Neale.*

'Tis the sublime of man—
Our noon-tide majesty—to know ourselves
Parts and proportions of one wondrous whole!
This fraternises man, this constitutes
Our charities and bearings. But 'tis God
Diffused through all, that doth make all One Whole.
—*S. T. Coleridge.*

**Reading.**

A holy life is the very gate of Heaven. But let us always remember that holiness does not consist in doing uncommon things, but in doing everything with purity of heart. It is made up of relative duties and of habitual devotion. Like the law of gravitation, which universally takes effect where not kept out by special counteraction, so it is with the cares, pleasures, labours, anxieties of life. Nothing but fellowship with God keeps them in check.—*Dr. Manning.*

The value of everything in life depends on its power to lead us to God by the shortest road.—*F. W. Faber.*

Neither pray I for these alone, but for them also which shall believe on ME through their word. That they shall all be one; as Thou, Father, are in ME, and I in THEE, that they may also be one in us.—*John* xvii. 20-21.

### Notes and Queries.

**The contents of the Editor's Letter-box have now reached such unwieldy proportions that it has become necessary to establish a hard and fast rule regarding Answers to Correspondents. In future, all questions requiring replies will continue to be answered in this column without any fee. If an answer is required by letter, a fee of half-a-crown must be enclosed with the note containing the enquiry. We are always pleased to help our numerous correspondents to the fullest extent, and we can trust them to sympathise in the overwhelming amount of writing which makes the new rules a necessity. Every communication must be accompanied by the writer's name and address, otherwise it will receive no attention.**

*Masseuse in Glasgow.*

(116) Will you tell me where I could qualify as a masseuse in Glasgow, and where I could take my certificate?—*Untrained Masseuse.*

If you write to the Secretary of the Society of Trained Masseuses, 12, Buckingham Street, Strand, she can give you the address of a lady who would train you for a fee of £5 5s.; you might afterwards attend the October examination of trained masseuses in London for your certificate.

*Probationer in Fever Hospital.*

(117) Can you tell me of any fever hospital where the matron would be willing to take a paying probationer for six months?—*L. C.*

Your quickest way to find what you want would be to insert an advertisement in our columns. There is an advertisement for a probationer in a fever hospital in "The Mirror" for June 11th.

*Superfluous Hairs.*

(118) Please tell me if Röntgen rays will destroy permanently and without injury "superfluous hairs" on face, and how and where I could have it done, or if there is any other cure.—*V. K.*

The only means of removing superfluous hair is by electrolysis. A skin specialist would be able to tell you to whom to go to have it done.

*Asylum or Hospital.*

(119) Please tell me of some good books on mental nursing suitable for a nurse preparatory to training. (2) I am anxious to train as nurse, but am slightly flat footed. Would training in an asylum be less trying than in a hospital? I should train with a view to taking up private work afterwards. (3) At what age do asylums take probationers?—*Would-Be Nurse.*

(1) The following books may be of service: "Nursing; its Theory and Practice," by Percy G. Lewis, M.D., price 3s. 6d.; and "Mental Nursing," by William Harding, M.D., price 2s. 6d. Both post free from Scientific Press, London. (2) Possibly asylum work might be lighter, but you must have three years' training in a general hospital in order to obtain your certificate. See carefully to your feet, wearing cashmere shoes, bathing the feet, and then rubbing them with dry alum. Lilley and Skinner are selling a contrivance called "Pond's Arch Support Socks" that might be of service to you. (3) Some asylums take probationers at nineteen. St Anne's Heath, Virginia Water, and the Northampton County Asylum are amongst the best training schools for mental nursing.

*Fees.*

(120) I am a certificated monthly nurse, but am now doing medical nursing, although not trained for it. What ought I to charge a week? My fees for monthly work are from 30s. to two guineas.—*Nurse H.*

We cannot recommend anyone to undertake such responsible work as nursing without having undergone a proper training.

*Training at the Temperance Hospital.*

(121) Could you tell me whether the training certificate offered at the Temperance Hospital, Hampstead Road, is considered good? Would the fact of it being a temperance hospital detract from the value of the certificate?—*America.*

The training is excellent at the London Temperance Hospital, and the fact that it is a temperance hospital in no way interferes with the value of the certificate.

*Qualifications of a Country Nurse.*

(122) Kindly inform me what qualifications are necessary for a nurse in a country district, also the best way to obtain such a post?—*C. F.* (Derby).

If you hold a two-year certificate, and are otherwise suitable, the Queen Victoria's Jubilee Nursing Institution will accept you as a Queen's Nurse, and give you six months' district training. If untrained, you may perhaps be accepted by some of the country associations to train for a cottage nurse. Lastly, see our advertisement columns.

*Society of Chartered Nurses.*

(123) Kindly tell me where I can find full information of the Society of Chartered Nurses?—*Accident.*

From the Secretary, 24, Princes Street, Cavendish Square, W.

*Free Maternity Training.*

(124) Would you tell me if there is any maternity hospital in which one could gain their certificate for monthly nursing free? If not, the cheapest way of obtaining one?—*Nurse Emma.*

There is no hospital that gives maternity training free. The cheapest training is to be gained at the East End Mothers' Home, 396, Commercial Road, E., of which you may get particulars from the secretary.

